# Nasopharyngeal Carcinoma and Its Microenvironment: Past, Current, and Future Perspectives

**DOI:** 10.3389/fonc.2022.840467

**Published:** 2022-03-02

**Authors:** Zhi Yi Su, Pui Yan Siak, Chee-Onn Leong, Shiau-Chuen Cheah

**Affiliations:** ^1^ Faculty of Medicine and Health Sciences, UCSI University, Kuala Lumpur, Malaysia; ^2^ Centre of Cancer and Stem Cells Research, International Medical University, Kuala Lumpur, Malaysia; ^3^ Institute for Research, Development and Innovation, International Medical University, Kuala Lumpur, Malaysia

**Keywords:** nasopharyngeal carcinoma, tumor microenvironment, Epstein-Barr virus, immunotherapy, exosomes

## Abstract

Nasopharyngeal carcinoma (NPC) is an epithelial malignancy that raises public health concerns in endemic countries. Despite breakthroughs in therapeutic strategies, late diagnosis and drug resistance often lead to unsatisfactory clinical outcomes in NPC patients. The tumor microenvironment (TME) is a complex niche consisting of tumor-associated cells, such as fibroblasts, endothelial cells, leukocytes, that influences tumor initiation, progression, invasion, and metastasis. Cells in the TME communicate through various mechanisms, of note, exosomes, ligand-receptor interactions, cytokines and chemokines are active players in the construction of TME, characterized by an abundance of immune infiltrates with suppressed immune activities. The NPC microenvironment serves as a target-rich niche for the discovery of potential promising predictive or diagnostic biomarkers and the development of therapeutic strategies. Thus, huge efforts have been made to exploit the role of the NPC microenvironment. The whole picture of the NPC microenvironment remains to be portrayed to understand the mechanisms underlying tumor biology and implement research into clinical practice. The current review discusses the recent insights into the role of TME in the development and progression of NPC which results in different clinical outcomes of patients. Clinical interventions with the use of TME components as potential biomarkers or therapeutic targets, their challenges, and future perspectives will be introduced. This review anticipates to provide insights to the researchers for future preclinical, translational and clinical research on the NPC microenvironment.

## Introduction

**Figure 1:**
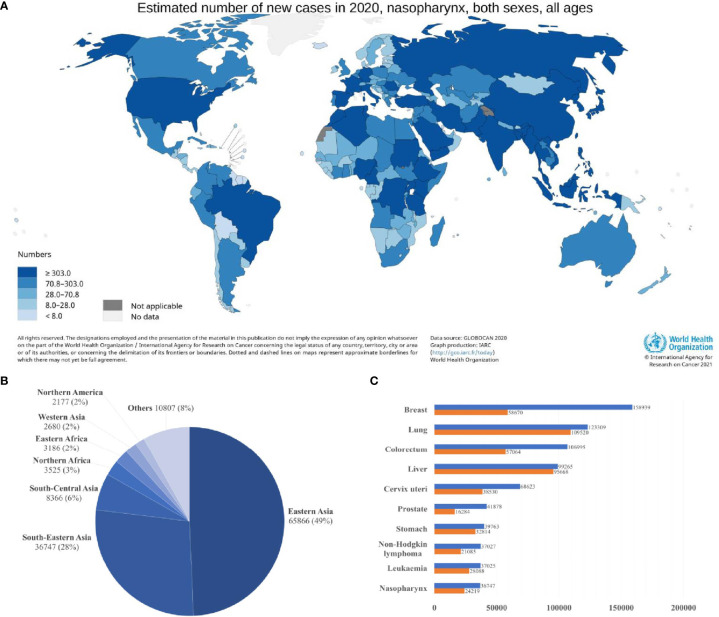
Global distribution of nasopharyngeal carcinoma **(A)** Estimated age-standardized incidence rate (ASR; world) **(B)** Estimated number of new cases in different world areas. **(C)** Estimated number of incident cases and deaths in South-Eastern Asia. Data source: GLOBOCAN 2020 (3).

Nasopharyngeal carcinoma (NPC) is a squamous cell neoplasm that originated from the nasopharyngeal mucosal lining, commonly found at the fossa of Rosenmüller (1, 2). In 2020, the International Agency for Research on Cancer (IARC) reported 133,354 incidences of NPC globally, which accounted for 0.7% of all cancers diagnosed (**Figure 1A**) (4). Intriguingly, NPC demonstrated a remarkable geographical distribution, where over 77% of NPC incidences were found in Eastern and South-Eastern Asia (**Figure 1B**). In South-Eastern Asia, NPC ranked the 10^th^ most common cancer among the entire population (**Figure 1C**) (3). Brunei had the highest age-standardized incidence rate of NPC (13.35 per 100,000 in males; 6.44 per 100,000 in females), followed by Maldives (10.67 per 100,000 in males; 3.3 per 100,000 in females), Indonesia (10.71 per 100,000 in males; 3.03 per 100,000 in females), Malaysia (9.53 per 100,000 in males; 3.05 per 100,000 in females) and Vietnam (8.12 per 100,000 in males; 2.79 per 100,000 in females) (5, 6). China had an age-standardized rate of 3.0 per 100,000, whereby the incidences are more prevalent among the Cantonese-speaking population which resided in southern China, including Guangzhou province (13.9 per 100,000 in males; 5.2 per 100,000 in females) and Hong Kong (12.8 per 100,000 in males; 4.0 per 100,000 in females).

The incidence of NPC is higher in males (96,371) than in females (36,983), with a ratio of about 2 to 3:1 (4). This phenomenon can be explained by the inheritance of genetic susceptibility associated with X chromosomes, leading to male predominance in the incidence of NPC (7). Sex hormones, such as estrogen, may play a protective role against NPC in females (8). Viral infection with Epstein-Barr virus (EBV) is the most common aetiologic factor in NPC, 100% of non-keratinizing NPCs are detected with EBV infection. Several environmental factors, including dietary consumption of preserved food, and social practice such as tobacco smoking and alcohol consumption, are associated with increased risk of NPC (1, 2, 9). Taken together, EBV infection, genetic and environmental factors contribute to the pathogenesis of NPC.

The application of intensity-modulated radiotherapy (IMRT) alone or with chemotherapy for treatment of NPC significantly improves the clinical outcome of NPC patients. NPC patients diagnosed with early-stage (stage I to II) have a 5-year overall survival (OS) as high as 94% (10–12). Tragically, despite the advances in medical treatment, over 60% of newly diagnosed patients have poor clinical outcomes as they are often diagnosed at late-stage, due to non-specific clinical presentation and lack of biomarkers for early detection (12). Early manifestations of NPC, such as headache, cervical mass, nasal obstruction, and epistaxis, are non-specific, leading to a misdiagnosis rate of approximately 43%, which ultimately results in delay in treatment and poor prognosis (10). When patients are diagnosed with late-stage (stage III-IV) NPC, the 5-year survival rate declines drastically, which is lower than 80% (10). The mortality risk of patients who were diagnosed with stage IV NPC is 3.41-fold higher in comparison with those diagnosed at early-stage (13). Other challenges include locoregional or distant recurrence, which affects 20 to 30% of patients, and therapeutic resistance (1, 12). Thus, it is imperative for the development of high specificity, high sensitivity, and non-invasive laboratory tests for the diagnosis and prognosis of NPC and the development of new therapeutic strategies to reduce morbidity and mortality of the disease.

**Figure 2:**
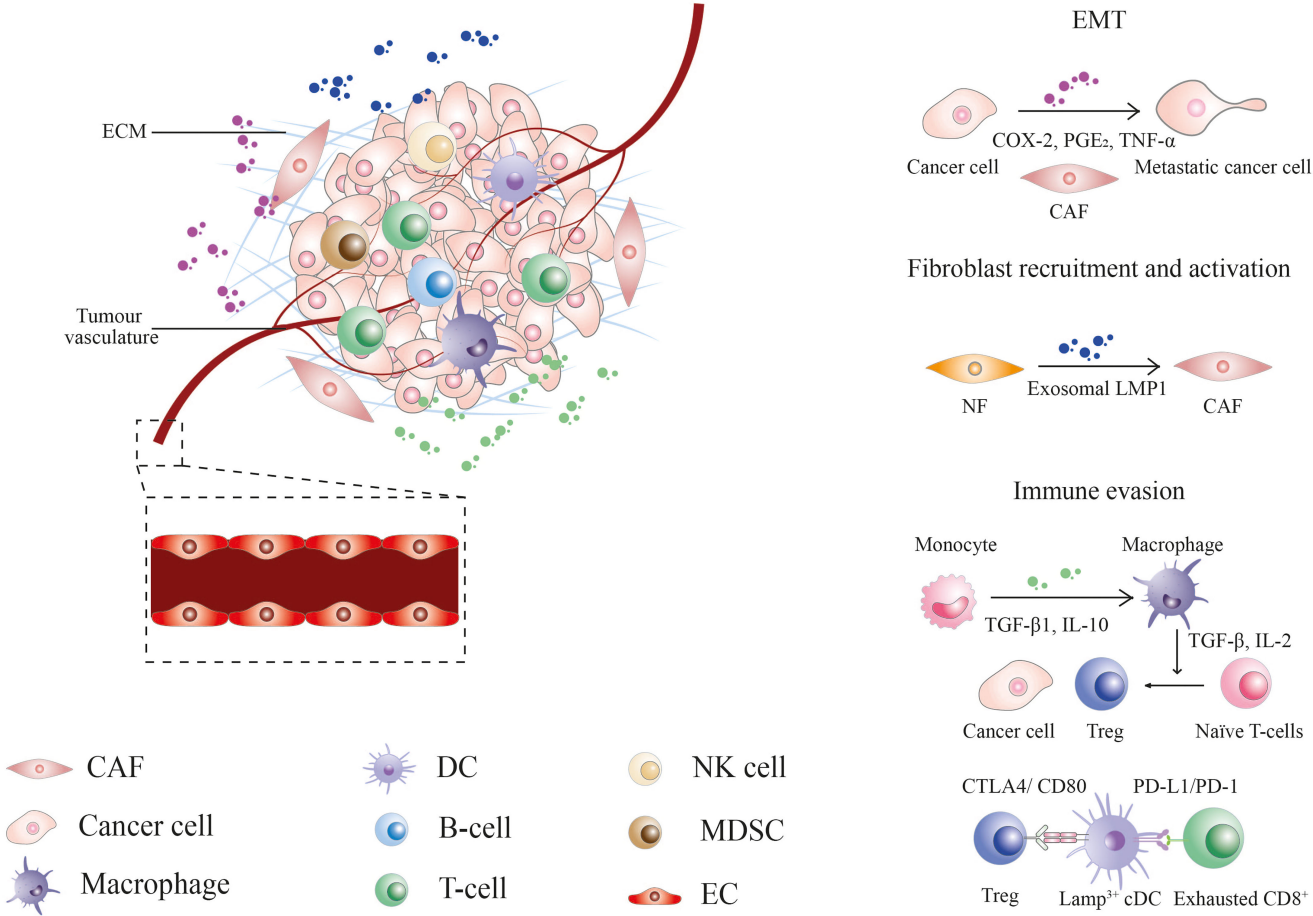
The nasopharyngeal carcinoma tumor microenvironment. CAF, cancer-associated fibroblast; COX-2, cyclooxygenase-2; CTLA4, cytotoxic T-lymphocyte associated protein 4; DC, dendritic cell; EC, endothelial cell; ECM, extracellular matrix; EMT, epithelial-mesenchymal transition; Exo-LMP, exosome-packaged latent membrane protein; IL, interleukin; MDSCs, myeloid-derived suppressor cells; NF, normal fibroblast; NK, natural killer; PD-1, programmed cell death protein 1; PD-L1, programmed death-ligand 1; PGE_2_, prostaglandin E2; TGF, tumor growth factor; TNF, tumor necrosis factor; Treg, regulatory T-cell.

The tumor microenvironment (TME) is a specialized niche made up of resident malignant and infiltrated cells, metabolites, and extracellular matrix, characterized by its heterogeneity and complexity (**Figure 2**). Stephen Paget’s “seed and soil” theory suggested the preference of tumor cells (the “seed”) to grow in a favourable microenvironment (the “soil”) (14). The Tissue Organization Field Theory supported the above hypothesis by suggesting that communication between the microenvironment and cell through biophysical and biochemical cues drives phenotypic transformation of cells, which eventually leads to cancer (15). The heterogenous TME further contributes to the progression of cancer by facilitating the acquisition of a series of hallmark capabilities, including [1] sustained proliferative signaling; [2] evasion of growth suppressors; [3] apoptotic resistance; [4] immortal replication; [5] angiogenesis; [6] invasion and metastasis; [7] reprogramming energy metabolism and [8] evasion from immune surveillance, as enumerated by Hanahan and Weinberg (16). These theories open a door towards a new era of medical research targeting the TME. Since the last few decades, several efforts have been made to explore the NPC microenvironment by immunohistological analysis of NPC biopsies. With the advancement of technologies, researchers can delineate the complex TME and cellular crosstalk at single-cell resolution, thereby facilitating the development of diagnostic, prognostic, and therapeutic tools against NPC (17–21).

As a requisite in tumor initiation, progression, and metastasis, the TME components and their interplay with cancer cells offer a variety of promising targets for the development of anti-cancer therapy against NPC. This review introduces the cellular and acellular players, their roles in the NPC microenvironment, current and potential biomarkers, and therapeutic strategies targeting TME.

## Components in Tumor Microenvironment

The NPC microenvironment is complex and highly heterogeneous, its components can be classified into cellular and acellular components. Cellular components include [1] tumor endothelial cells (TECs), which forms blood and lymphatic vascular networks for transportation of nutrients and oxygen, [2] cancer-associated fibroblasts (CAFs), which support tumor growth, survival, invasion, and migration, and [3] immune cells, which are involved in immune reactions in the TME (22–25). On the other hand, acellular components include the extracellular matrix (ECM), which facilitates tumor development, progression, and metastasis (26, 27).

### Tumor-Infiltrating Immune Cells

The NPC microenvironment is characterized by the intense filtration of tumor-infiltrating immune cells (TIICs), which constitute 40 – 50% of the tumor mass in NPC, with EBV-negative CD3^+^ T-lymphocytes as the commonest infiltrate (28–30). Despite its abundance, lack of effective immune response, and the presence of immunosuppressive infiltrates such as regulatory T-cells (Tregs), M2 macrophages, and myeloid-derived suppressor cells (MDSCs) leads to immunotolerance, promoting tumor progression. TIICs can be further classified based on lymphoid and myeloid lineage. Tumor-infiltrating lymphocytes (TILs), including T-cells and B-cells, are abundant in the NPC microenvironment as a result of an immune response against EBV. On the other hand, tumor-infiltrating myeloid cells (TIMs) consist of various cell types, including mature mast cells, monocytes and macrophages, dendritic cells (DCs), and pathologically activated immature MDSCs (17, 31, 32).

#### Tumor-Infiltrating Lymphocytes

TILs, including T-cells and B-cells, are most predominant in the NPC microenvironment. Tumor-associated T-cells consisted of several subpopulations, namely naïve T-cells, cytotoxic T-cells (CTLs), exhausted T-cells, and Tregs (17–21). CD3^+^ T-lymphocytes, which are comprised of CD4^+^ T-helper cells and CD8^+^ T-suppressor cells, is the predominant lymphocytes infiltrating the NPC microenvironment. The majority of these lymphocytes express the activation marker OKT10, however, restriction on human leukocyte antigen (HLA) and T-cell receptor gene expression might contribute to tumor evasion from immune surveillance (29, 30, 33, 34). Consistent with these findings, transcriptomic studies reported that CD4^+^ and CD8^+^ T-cells clusters in NPC are highly activated and exhausted, as they co-express exhaustion markers (*LAG3*, *TIGIT*, *PDCD1*, *HAVCR2*, *CTLA4*, *TOX*) and effector molecules (*GZMB*, *GZMK*, *INFG*, *NKG7*, *GNLY*, and *IL2*) (17–21). Exhausted T-cell clusters display intermediate-to-high cytotoxic activity, implying a dynamic transitional process from activated T-cells to exhausted T-cells (17). Both intrinsic and extrinsic mechanisms are involved in the exhaustion or dysfunction of T-cells. The intrinsic mechanisms include [1] expression of inhibitory receptors, including programmed cell death protein 1 (PD-1), cytotoxic T-lymphocyte-associated protein 4 (CTLA-4), T-cell immunoglobulin and mucin-domain containing-3 (TIM-3), lymphocyte activation gene (LAG-3), B and T lymphocyte attenuator (BTLA) and T-cell immunoglobulin and ITIM domain (TIGIT), [2] decreased cytokine signaling pathways which suppresses the production of interleukin (IL)-2, tumor necrosis factor (TNF)-α, interferon (IFN)-γ and granzyme B (GzmB) in a hierarchical manner, and [3] transcription factors such as the nuclear factor of activated T-cells 1 (NFATC1) (20, 35). Extrinsic mechanisms, on the other hand, include [1] inhibitory effects exerted by regulatory cells including Treg cells, DCs, tumor-associated macrophages (TAMs), MDSCs, [2] immunosuppressive cytokines, such as transforming growth factor (TGF)-β and IL-10, and [3] interaction with major histocompatibility complex (MHC) class II-expressing NPC cells (18, 35).

Tregs are immunosuppressive cells expressing CD4^+^CD25^high^Foxp3^+^ markers (18, 36). Tregs mediate tumor escape from immune surveillance by [1] involvement of immune checkpoint molecules, such as PD-1 and CTLA-4, [2] secretion of cytokines, for instance, IL-10 and TGF-β, which elicits inhibitory effects on immunoreactive cell proliferation, [3] induction of T-cells apoptosis through secretion of cytotoxic perforin and GzmB or ligand-receptor interaction through Fas/FasL, [4] cell contact-dependent suppression of naïve T-cells proliferation, [5] metabolic modulation through enhancing immunosuppressive metabolites, such as indoleamine 2,3-dioxygenase (IDO), in the TME (37–39). Studies revealed enhanced expression level of *LGALS1* in NPC-derived Tregs, which was reported to mediate immunosuppression by upregulation of cell-surface programmed death-ligand 1 (PD-L1) and galectin-9 (Gal-9) in head and neck cancer (18, 40). Physical or pharmacologic depletion of Tregs targeting drugs removes their suppression effect and restores EBV-specific CD8^+^ T-cells (41).

NK cells are the first line of defense against viral infections and neoplasms. Studies reported diminished expression of inhibitory surface receptors of NK cells, including NK group 2, member D (NKG2D), NKp30, and NKp46 as a mechanism for immune evasion. Expression of *metastasis-associated in colon cancer-1* (*MACC1*) gene on NPC cells and enhanced level of soluble MHC class I chain-related molecular A (MICA) is correlated with the downregulation of NKG2D surface expression on NK cells, contributing to tumor progression (42–44). Moreover, the expression of inhibitory receptors such as IL-18-induced PD-1 also mitigates the anti-tumor effect of NK cells (45). Paradoxically, in a recent transcriptomic study of NPC-infiltrating cells, Gong et al. reported expression of cytotoxic genes and chemokine encoding genes in NK cells of the NPC microenvironment, indicating their role as a positive regulator of the immune response against the malignancy (17).

B-cells represent the second largest and most diverse cells in the NPC microenvironment. In particular, EBV positivity is correlated with an increase in B-cell abundance and diversity. The various phenotypes of tumor-infiltrating B-cells include memory B-cells, germinal center B-cells, plasmablast-like cells and plasma cells. B-cells are recruited into tertiary lymphoid structures by tumor-derived PD-1^+^ exhausted CD4^+^ T- cells through the CXCL13/CXCR5 axis (20, 46, 47). CD19^+^ B-cells positively correlated with better prognosis in EBV-positive NPC patients (48). Consistent with this finding, higher expression of B-cell-associated gene signatures such as *CD79A*, *MS4A1*, *IGHD* and *FCRL4* favored the survival in NPC patients, suggesting potential anti-NPC immunity by NPC-infiltrating B-cells (17). In contrast, IL-10^+^ B-cells are immunosuppressive B-cells induced by NPC-derived miRNA-21 (49).

#### Tumor-Infiltrating Myeloid Cells

MDSCs, macrophages and DCs are the three major myeloid-lineage subtypes in the NPC microenvironment. Under the influence of tumors, alteration of myelopoiesis and impairment of progenitor cells differentiation leads to accumulation of MDSCs. Metabolic alteration by EBV-encoded latent membrane protein (LMP)1 enhances the secretion of IL-1β, IL-16 and GM-CSF through NLRP3 inflammasome, cyclooxygenase-2 (COX-2) and P-p65, which subsequently activates signal transducer and activator of transcription (STAT)-3 signaling pathway to induce MDSCs accumulation (50, 51). Many signature genes of MDSCs are associated with exhaustion and cell cycle arrest of immune cells, implying their role in the induction of immunosuppression through inhibition of T-cell proliferation, promotion of T-cell anergy, and induction of T-cell apoptosis (17, 51). Other than that, MDSCs directly promote NPC cell migration, invasion, and metastasis *via* contact-dependent induction of epithelial-mesenchymal transition (EMT) in NPC cells *in vitro* through upregulation of *COX-2* expression and activation of *β-catenin/TCF4* pathway. Clinically, HLA-DR^-^CD33^+^ MDSCs and *COX-2* predict poor disease-free survival (DFS) in NPC patients (32).

Monocytes and macrophages are the most predominant cell types in TIMs, which account for around 50% of TIMs in NPC (52). Macrophages may replace T-lymphocytes in anti-tumor immune response and preventing lymphatic spread (53). Macrophages demonstrate a high degree of plasticity when exposed to signals from the TME (52). They can be classically polarized into inflammatory M1 macrophages or activated into immunosuppressive M2 macrophages (54). NPC cells induce polarization of CD163^+^ M2 macrophages *via* TGF-β1 and IL-10, which subsequently recruits Foxp3^+^ Tregs through induction of conversion from naïve T-cells, *via* TGF-β and IL-2, and chemotaxis, leading to immune escape. Tregs, in return, secrete TGF-β1 and IL-10 to promote M2 macrophage differentiation, forming a positive feedback loop that favors immune escape in NPC (55). Single-cell transcriptomic analysis *via* RNA sequencing reported co-expression of M1 and M2 gene signatures in NPC-derived TAMs, suggesting an M1-M2 coupled activation pattern in TMEs which gives rise to a unique phenotype that exhibits both pro-inflammatory and pro-tumoral functions (18). The potential anti-tumor capacity of NPC-derived macrophage is exhibited by its high expression of CXC chemokine ligands CXCL9 and CXCL10 which recruit CXCR3^+^ NK cells and CD8^+^ T-cells into the TME (20). In contrast, pro-tumorigenic TAMs display expression of angiogenic signature *SPP1*, which is typically associated with poor prognosis (31). Furthermore, exosomal miR-18a derived from M2 macrophages promote NPC progression, invasion, and tumor growth in *in vitro* and *in vivo* animal models *via* TGF-β signaling pathway by repression of transforming growth factor-beta III receptor (TGFBR3) (56).

DCs initiate antigen-specific immune responses by recognition and presentation of antigen to T-cells. Early studies reported the presence of T-zone histiocytes such as DCs in about half of NPC tissues, their densities significantly correlates with favorable prognosis in NPC patients, implying their role in anti-tumor immunity (57, 58). Tumor-infiltrating DCs can be immunogenic or regulatory, depending on the environmental signals. Different subtypes of DCs are present in the TME, for instance, classical dendritic cells (cDCs) and plasmacytoid dendritic cells (pDCs). The proportion of pDCs is high in NPC compared to other malignancies. pDCs are associated with favorable prognostic value, which implies their potential roles in the induction of anti-tumor immune response. Consistent with this finding, *GZMB*, a gene that encodes pro-apoptotic enzyme GzmB, is highly expressed in pDCs (20). On the other hand, cDCs can be further divided into three distinct subsets, including classical *CLEC9A^+^* cDC1s and *CDC1C^+^* cDC2s and a mature phenotype *LAMP3^+^* cDCs. *LAMP3^+^* cDCs can be derived from both cDC1s and cDC2s, resulting in *LAMP3^+^* cDCs with different transcriptomic properties and might exhibit diverse functions. *LAMP3^+^* cDCs represent a group of regulatory DCs demonstrating a high level of differentiation and apoptosis but low antigen presentation, with elevated expression of immune-suppressive related genes, such as *CD274* (PD-L1), *PDCD1LG2* (PD-L2), *IDO1*, and *CD200*. These cells potentially exert regulatory activities *via* recruitment of Tregs, secretion of immunosuppressive molecules, and ligand-receptor inhibition of T-cell activities (21, 31).

Intercellular interactions among LAMP3^+^ DCs, Treg cells, exhausted CD8^+^ T-cells, and malignant cells nurture an immunosuppressive NPC microenvironment. LAMP3^+^ DCs potentially recruit peripheral Tregs through C-C motif chemokine ligand (CCL)/C-C motif chemokine receptor (CCR) interactions such as the CCL17/CCR4 and CCL22/CCR4 signalling pathways. Furthermore, LAMP3^+^ DCs elicit suppressive activities on CD8^+^ T-cells *via* CD200/CD200R signalling and PD-L1/PD-1, leading to the exhaustion of CD8^+^ T-cells. On the other hand, Treg cells expressing CTLA-4 interacts with CD80/CD86 on LAMP3^+^ DCs, whereby the interaction might restrain the antigen presentation of DCs and promote their secretion of IDO1 to induce the proliferation of Treg cells (21).

The proportion of mast cells is relatively higher in NPC when compared to other malignancies. NPC-derived mast cells have a high anti-tumor *TNF* to angiogenic *VEGFA* ratio, implying high anti-tumor capacity, whereas mast cells in other malignancies are generally pro-tumorigenic. Hence, mast cells are correlated with a survival advantage in NPC. The anti-tumor phenotype of mast cells is possibly driven by the presence of *IL1B^+^* macrophages *via* IL1B/ADRB2 ligand-receptor interaction (31, 59).

### Cancer-Associated Fibroblasts

CAFs, also termed tumor-associated fibroblasts, are found abundantly in the cancer stroma. CAFs have elongated spindle morphology, displayed mesenchymal biomarkers, such as alpha-smooth muscle actin (α-SMA), platelet-derived growth factor receptors (PDGFRs), vimentin and fibroblast activation protein (FAP), and lack of genetic mutations (60, 61). The CAFs predecessors are recruited from diverse origins, which include resident tissue fibroblasts, peri-tumoral adipocytes, endothelial cells through endothelial-mesenchymal transition (EndMT), epithelial cells through EMT, bone-marrow-derived mesenchymal stem cells and haematopoietic stem cells (25, 60, 62). In NPC, extracellular vesicle-packaged EBV-encoded LMP1 can promote the activation of normal fibroblasts into CAFs *via* the nuclear factor-kappa B (NF-κB) p65 pathway (63). α-SMA expression in CAFs predicted an adverse prognosis in patients with NPC. A high density of CAFs is correlated with shorter OS and lower 5-year survival rates, suggesting CAF density as an independent prognostic factor for NPC patients, suggesting their role in tumorigenesis (64). Consistent with this finding, several studies reported that CAFs promote neoangiogenesis, metastasis, and therapeutic resistance in NPC (65–67).

CAFs stimulate neoangiogenesis in NPC. Genes correlated with endothelial cells abundance were highly expressed by fibroblasts, implying their potential roles in endothelial cell recruitment and angiogenesis (18). The immunohistochemical method revealed high expression of α-SMA fibroblasts in NPC stroma, together with increased intensities of the chemokine stroma-derived factor-1 (SDF-1, also known as CXCL12), a mediator of the recruitment of endothelial progenitor cells, and its receptor CXCR4 in NPC cells. Vascular endothelial growth factor (VEGF) was detected in both cancer and stromal cells, indicating secretion of a significant amount of VEGF in these cells. Prominin 1 (PROM 1, or CD133) and VEGF receptor (VEGFR)-2 double-positive endothelial progenitor cells or CD34 positive cells were observed in the stroma, suggesting tumor-associated neoangiogenesis. Statistical analyses revealed a positive correlation between α-SMA and endothelial antigens CD34, suggesting that CAFs and NPC tumor cells may enhance neoangiogenesis in a VEGF- and SDF-1-dependent manner by the recruitment endothelial progenitor cells from the bone marrow into the tumor stroma (65).

CAFs support tumor metastasis in NPC. Increased density of α-SMA-expressing CAFs at metastatic sites of NPC compared with primary sites, along with upregulation of COX-2 or prostaglandin-endoperoxide synthase (PTGS2) in CAFs, indicated the involvement of fibroblasts and COX-2 in NPC metastasis. High expression of COX-2 catalysed CAF-secreted prostaglandin E2 (PGE2), which induces EMT, thereby promoting NPC cell migration and invasiveness *in vitro*. Furthermore, COX-2 in host fibroblasts promotes lung metastasis and correlated with the expression of TNF-α expression in mouse models, suggesting that high expression of COX-2 in fibroblasts promotes NPC metastasis through the COX-2-PGE2-TNF-α axis. Consistent with this finding, the expression of COX-2 in CAF was positively correlated with N stage, relapse, and poor survival in patients with NPC (66).

CAFs promote therapeutic resistance and immune evasion. CAFs induced the formation of radioresistance and promoted NPC cell survival following irradiation *via* the IL-8/NF-κB pathway to reduce irradiation-induced DNA damage. Moreover, CAFs express immunosuppressive factor *IDO1*, which encodes the enzyme IDO. IDO catalyses the production of L-kynurenine (Kyn), which subsequently promote the generation or differentiation of tolerogenic immune cells by interacting with aryl hydrocarbon receptors on immune cells. High expression of IDO is inversely correlated with the density of CD3^+^ T-cells and predicted poor survival outcomes in NPC patients. Hence, CAFs may promote immune suppression in NPC by the expression of IDO (18, 68).

CAFs exhibit a supportive role in tumor progression through the remodeling of the ECM (25). NPC-derived fibroblasts express genes that encode ECM components, including *COL1A1*, *COL1A2*, *LUM*, *FN1*. This suggests the complexity of ECM in the NPC microenvironment and the possible interaction with tumors and stromal cells *via* integrin signaling, indicating integrin receptors on tumor and immune cells as potential therapeutic targets for disruption of ECM-dependent tumor progression and suppressive immunomodulation (17). Increased production and crosslinking of collagen, such as COL1A1, increase the stiffness of ECM, leading to the promotion of tumor progression through increased integrin signaling (25, 27). On the other hand, fibronectin 1 (FN1) is shown to increase migration and invasion of NPC cells by upregulation of matrix metalloproteinase 9 (MMP9) and MMP2, which are ECM-digesting enzymes mainly produced by CAFs. FN1 also suppresses NPC cell apoptosis *via* the NF-κB pathway by upregulation of the expression of BCL2 and P65 (25, 69). CAFs mediated directional migration of cancer cells by assembling a fibronectin-rich ECM with anisotropic fiber orientation through increased non-muscle myosin II- and PDGFRα-mediated contractility and traction forces (62, 70).

### Angiogenesis, Tumor Endothelial Cells and Hypoxia

Angiogenesis, the formation of blood vessels from existing vasculature in response to a hypoxic condition, is one of the hallmarks of cancer that contributes to tumor growth, development and metastases (71). The vascular niche is associated with stem cell-like NPC at the invasive front (72). The tumor vasculature is lined internally by TECs and surrounded externally by perivascular cells. TECs are characterized by their genetic instability, which contributes to the development of distinct phenotypes that promote therapeutic resistance, tumor progression and metastasis. TECs also interact with tumor cells by secretion of angiocrine factors, contributing to tumor migration and metastasis (73, 74). Drug-resistant human microvasculature endothelial cells (HMECs) are reported to promote progression, EMT and chemoresistance in NPC through secretion of exosomes (75). In NPC, TECs demonstrated enhanced capacity to recruit CTLs and Tregs *via* CXCL9–CXCR3 and CXCL10–CXCR3 while simultaneously inhibiting CTLs through PDL2–PD1 interaction, suggesting their role in the mediation of an immunosuppressive niche in the NPC microenvironment (18).

Hypoxia was detected in over 80% of NPC tumors as a result of the high requirement of oxygen due to increased proliferation and metabolism. It was reported to play an important role in NPC by regulating its apoptotic activity, metastasis, angiogenesis, metabolic adaptation, and therapeutic resistance, thereby representing a potential target for the treatment of NPC. Hypoxic stress prevents cell survival by inducing the upregulation of anti-apoptotic proteins such as growth arrest and DNA-damage-inducible beta (Gadd45β) and immediate early response 3 (IER3) (76). Hypoxia promotes tumor cell migration by EMT stimulation and ECM remodeling through Notch signaling (27). Lysyl oxidase (LOX), an extracellular matrix-remodeling enzyme, was significantly upregulated in hypoxic conditions and predicted poor prognosis in patients with NPC (76, 77). In addition, hypoxia induces the upregulation of VEGF, which is responsible for neoangiogenesis. Furthermore, hypoxia mediates metabolic adaptation and acidosis in NPC (76). A metabolic shift in NPC infiltrating lymphocytes was reported to induce the exhausted phenotype of T-cells through overexpression of miR-24. miR-24 overexpression suppresses the expression of MYC and FG11 in TILs and disrupts MFN1-mediated mitochondrial fusion, thereby inducing T-cell exhaustion (78).

Various molecular signals and tumor-derived factors are involved in angiogenesis. VEGF is a pro-angiogenic factor that activates and stimulates proliferation and migration in VEGFR expressing endothelial cells. VEGF also alters vascular permeability by loosening the junctions between endothelial cells, favouring tumor intravasation (79). Besides hypoxia, increased VEGF production can be induced by EBV-encoded LMP1 through COX-2 expression in NPC cells (80). High expression of tissue VEGF is associated with reduced OS and DFS in NPC patients (81).

## Intercellular Communications in Nasopharyngeal Carcinoma Microenvironment

Intercellular communication between malignant and stromal cells in the NPC microenvironment is established *via* paracrine mechanisms involving cytokines and chemokines, extracellular vesicles including exosomes and ligand-receptor interactions. The NPC microenvironment is primarily comprised of heavy immune infiltrates, with CD3^+^ T-lymphocytes as the most abundant lymphocyte infiltrates in NPC biopsies (37, 82). EBV-encoded proteins, such as LMP1, initiate immune cell recruitment by the regulation of multiple signalling pathways associated with cytokine and chemokine secretion from tumor and immune cells. Immunosuppression, mediated by cytokines and regulatory cells, facilitates malignant cells to escape from anti-tumor immune response and promotes tumor growth and progression. Tumor-derived exosomes, carrying viral proteins and immunosuppressive substances, and ligand-receptor interactions, such as PD-1/PD-L1 signalling pathway, also contribute to the construction of an immunosuppressive TME (28, 83).

### Chemokines and Cytokines

EBV-infected nasopharyngeal epithelial cells construct the NPC microenvironment, which is characterized by heavy infiltration of immune cells with suppressed immune activities, by secretion of EBV-encoded products that activate several inflammatory-associated signalling pathways. A variety of cytokines, such as IL-6, IL-8, interferon-inducible protein 10 (IP-10), TNF-α, VEGF, and macrophage inflammatory protein (MIP)-3α are elevated in NPC patients (84). LMP1 recruit immune cells into the tumor site by upregulating several cytokines through NF-κB and STAT3 signaling pathways (82, 85). These include regulatory cells with immunosuppressive influence in the TME. NPC cells induce polarization of macrophage into immunosuppressive M2-like phenotype through secretion of interferon-stimulated gene 15 (ISG15) (18). EBV nuclear antigen 1 (EBNA1) induce the production of CCL20, or MIP-3α to promote chemotaxis of Tregs to the tumor site (86, 87). In addition, EBV-encoded small RNAs (EBERs) activates the toll-like receptor 3 (TLR3) pathway to produce inflammatory cytokines such as CXCL8 which recruit and activate TAMs. The inflammatory responses in NPC cells are amplified by a positive feedback loop consisting of EBER, LMP1 and NF-κB (88). Metabolic reprogramming by LMP1 leads to alteration in Nod-like receptor family protein 3 (NLRP3) inflammasome, COX-2 and P-p65 signaling pathways, which results in the release of cytokines, including IL-1β, IL-6 and granulocyte-macrophage colony-stimulating factor (GM-CSF), contributing to the expansion of MDSCs (50). Stromal cells, including TECs, can attract CTLs and Tregs *via* chemokine-receptor interactions such as CXCL9/CXCR3 and CXCL10/CXCR3, however, cytotoxic activities of CTLs are inhibited by PD-L2/PD1 interaction (18).

Chemokines and cytokines facilitate tumor elusion from immune surveillance. EBER, LMP1 and EBV lytic transactivator, Zta, are positively correlated with the production of IL-10. BCRF1, or viral IL-10, share immunosuppressive properties with human IL-10. IL-10 induces immunosuppressive activities by inhibition of antigen-specific T-cell proliferation, induction of T-cell apoptosis and inhibition of IFN-γ secretion from NK cells. LMP1 upregulates IL-18 and IP-10 (CXCL10), which take part in the recruitment and immunosuppression of CXCR3^+^ NK cells and T-cells (45, 82, 85, 89). IL-6 and TNF-α mediate the expression of IDO, which demonstrates an immunosuppressive role by suppression of T-cell proliferation and impairment of CD8^+^ T-cells cytotoxic activities. Pre-treatment serum levels of IL-6 and TNF-α are negatively correlated with the 2-year survival rate in NPC patients (90, 91). Increased levels of IL-18 in NPC induces NK cells exhaustion *via* upregulation of expression of PD-1 on NK cells (45).

Furthermore, chemokines induce metastasis and promote tumor progression. IL-8 induce EMT by activating AKT signalling, enhancing migration and invasion of NPC cells. Enhanced level of IL-8 predicts adverse OS, DFS, and DMFS of NPC patients (92).

### Exosomes

Exosomes are small (30-100 nm) extracellular vesicles that originated from the plasma membrane. They are often involved in the intercellular interactions in the TME, influencing various cellular processes, including cell growth, angiogenesis, EMT, metastasis, immune tolerance, and therapeutic resistance (93). Exosomes may play a significant role in the formation of a premetastatic milieu by acting as a vehicle for tumor-derived factors that modulate pre-metastatic sites (15). Exosome contents include transcription factors, enzymes, ECM proteins, lipids, and nucleic acids, such as DNA, mRNA, and non-coding RNA. Based on their parental cells, exosomes in NPC can be classified into nasopharyngeal carcinoma-derived exosomes (NPC-Exo) and EBV-related exosomes (94–96).

#### NPC-Derived Exosomes

NPC-derived exosomes (NPC-Exos) [1] mediate the tumor immune microenvironment, [2] enhance angiogenesis, [3] induce EMT and [4] promotes resistance towards chemoradiotherapy as summarized in **Tables 1** and **2**. NPC-Exo facilitates tumor progression by promoting tumor evasion from immune surveillance. NPC-Exo carries clusters of microRNAs (miRNAs) associated with the downregulation of the MARK1 signaling pathway, including miR-24-3p, miR-891a, miR-106a-5p, miR-20a-5p, and miR-1908, leading to altered T-cell proliferation and differentiation. Hypoxia-induced exosomal miR-24-3p modulates the phosphorylation of ERK and STAT protein by repression of FG11 expression, leading to impeded T-cell proliferation, induction of Foxp3^+^ Tregs differentiation and inhibition of Th1 and Th17 differentiation. Low levels of exosomal serum miR-24-3p and high FGF11 expression predicts favorable DFS in NPC patients and may serve as potential prognostic biomarkers in NPC. Furthermore, NPC-Exo enhances the levels of pro-inflammatory cytokines IL-1β, IL-6 and IL-10 and reduce the levels of IFN-γ, IL-2 and IL-17 in CD4^+^ and CD8^+^ T-cells (113, 114, 125). Exosome-packaged CCL20 expands the Tregs population by promoting Tregs recruitment and conversion of conventional T-cells into inhibitory Tregs in the TME. CCL20 also enhances the suppressive effect of Tregs by inducing the overexpression of *TNFRSF4*, *SELL*, *ICAM1*, *TBX21*, *CCR6*, *TNF*, *GZMB*, *TGFB1*, *IL10*, *IL2* and *IL15*, which are associated with Treg phenotype, properties, and recruitment capacity (107, 113). Moreover, exosomes also transport Gal-9, which is associated with myeloid lineage-mediated immunosuppression through regulation of the expression of pro-inflammatory cytokines for the expansion of MDSCs by attenuating STING signaling (100).

**Table 1:** Exosomal content and their functions.

Class	Exosomal content	Function	Ref
Protein	HAX-1	Tumor growth, angiogenesis	(97)
	HMGB3	Angiogenesis, metastasis	(98)
	ICAM-1, CD44v5	Angiogenesis	(99)
	Gal-9	Immunosuppression	(100)
	HIF-1α	Metastasis	(101)
	DDX53	Chemoresistance	
lncRNA	CCAT2	Angiogenesis	(102)
circRNA	circMYC	Radio-resistance, cell proliferation	(103)
Enzyme	PFKFB3	Angiogenesis, tumor proliferation and metastasis	(104)
	MMP13	Invasion and metastasis	(105, 106)
Chemokine	CCL20	Immunosuppression	(107)
miRNA	miR-17-5p, miR-23a, miR-BART-10-5p, miR-18a, miR-144	Pro-angiogenesis	(108–111)
	miR-9	Anti-angiogenesis	(112)
	miR-24-3p, miR-891a, miR-106a-5p, miR-20a-5p, miR-1908	Immune regulation	(113, 114)
	miR-301a-3p	Metastasis	(115)
	miR-34c, miR-433-3p		(116, 117)

**Table 2:** Clinical studies targeting NPC microenvironment.

Target	Ref	Study	Conditions	Phases
Immunotherapy				
CTLA-4 & PD-1	NCT04220307	AK104	Metastatic NPC	II
	NCT04945421	IBI310 & Siltilimab	Anti-PD-1/PD-L1 Resistance R/M NPC	I/II
	NCT02834013 NCT03097939	Ipilimumab & nivolumab	Rare tumors, including NPC	II
EBV	NCT03648697	EBV-TCR-T (YT-E001) cells	EBV-positive R/M NPC	II
	NCT02287311	LMP, BARF1 & EBNA1 Specific CTL	R/M NPC	I
	NCT02578641 (118)	EBV-specific CTL & Chemotherapy	Advanced NPC	III
LMP2	NCT03925896	LMP2 Antigen-specific TCR T-cell Therapy	R/M NPC	I
PD-1	NCT03707509	Camrelizumab & Chemotherapy	R/M NPC	III
	NCT04944914	Camrelizumab & Stereotactic Body RT	R/M NPC	III
	NCT04978012	Camrelizumab & Fluzoparib	NPC	II
	NCT04833257	Chemotherapy & Tislelizumab	LA-NPC	II
	NCT04447612	Durvalumab & chemoradiation	R/M, platinum-resistant NPC	II
	NCT02339558 (119)	Nivolumab	R/M NPC	II
	NCT03267498	Nivolumab & Chemoradiation	Stage II - IVB NPC	II
	NCT03544099	Pembrolizumab	NPC	II
	NCT03734809	Pembrolizumab & Chemoradiation	NPC	II
	NCT04736810	Penpulimab Combination Therapy	NPC	II
	NCT03558191	SHR-1210	R/M NPC	II
	NCT04917770	Sintilimab & Multimodal RT	NPC	II
	NCT04376866	Toripalimab	Recurrent NPC	III
	NCT03925090	Toripalimab & CCRT	NPC	II
	NCT04534855	Treprilimab	Recurrent NPC	II
	NCT04421469	Triprilimab (JS001) & Chemotherapy	NPC	II
PD-1 & EBV	NCT03044743	PD-1 knockout EBV-CTLs	Advanced EBV-associated malignancies	I/II
PD-L1	NCT04282070	SHR-1701	R/M NPC	I
PD-L1 & VEGFR-2	NCT05020925	SHR-1701 & Famitinib	NPC	I/II
TGF-β	NCT02065362	TGF-β Resistant CTLs	EBV-positive NPC	I
TIM-3	NCT02817633	TSR-022 (combolimab)	Advanced solid tumors	I
T-cells	NCT04476641	DC-CIK Immunotherapy	Solid tumors, including NPC	II
Anti-angiogenic therapy				
VEGF	NCT00408694 (120)	Bevacizumab & Chemoradiation	LA NPC	II
VEGF/VEGFR signaling	NCT02636231	Endostar	Recurrent NPC	II
	NCT04447326 (121)	Endostar & Chemotherapy	LA-NPC	II
	NCT03932266		NPC	II
VEGFR	NCT03639467	Anlotinib & Gemcitabine/Cisplatin	R/M NPC	Ib/II
	NCT01462474 (122)	Famitinib & chemoradiation	LA NPC	I
	NCT00454142 (123)	Pazopanib	R/M NPC	II
	NCT00747799 (124)	Sorafenib & chemotherapy	R/M NPC	II
VEGFR & PD-L1	NCT04562441	Axitinib & Avelumab	R/M NPC	II

R/M, recurrent/metastatic; LA, locally advance.

Exosomes mediate angiogenesis by the transportation of pro-angiogenic and angiogenic-suppressive proteins and miRNAs, which mediate multiple angiogenesis-associated pathways (99). miR-17-5p and miR-23a are angiogenesis promoters, targeting BAMBI and testis-specific gene antigen (TSGA10) respectively (108, 109). In contrast, miR-9 inhibits angiogenesis by downregulating MDK and regulating PDK/Akt pathway.

Exosomes are also involved in the promotion of tumor invasion and metastasis. For instance, miR-301a-3p, which targets B-cell translocation gene 1 (*BTG1*) mRNA, promotes proliferation, migration, invasion and EMT of NPC (115). Under hypoxic condition, exosomes containing MMP13promotes metastasis by inducing EMT of malignant cells or mediating the TME by interacting with stromal fibroblasts and endothelial cells, hence promoting tumor invasion (105, 106).

Exosomes contributed to the development of resistance towards chemotherapy and radiotherapy. Taxol-resistant NPC cells can transfer dead-box helicase 53 (DDX53) into normal NPC cells to promote resistance to Taxol through upregulation of multidrug resistance 1 (MDR1) (126). circMYC is a circulating exosomal circular RNA (circRNA) associated with cell survival from radiotherapy. circMYC could sponge tumor suppressing miR-20b-5p and let-7e-5p, hence influencing their downstream targets, argonaute RISC component 1 (AGO1) and cryptochrome circadian regulator 2 (CRY2), affecting tumor progression (103).

#### EBV-Related Exosomes

EBV-infected NPC cells promote tumor growth by transferring viral oncoprotein such as LMP1, signal transduction molecules, and virus-encoded miRNA through exosomes (127). It is reported that recipient cells internalized exosomes derived from EBV-infected cells *via* caveola-dependent endocytosis (128).

LMP1 is an EBV-encoded gene product that is detected in almost all primary NPC specimens. A small amount of LMP1 facilitates tumor progression, whereas high LMP1 expression induced growth inhibition and cell apoptosis. LMP1 exerts its oncogenic activity by activating several signaling cascades including NF-κB, PI3K-AKT, ERK-MAPK, JNK, JAK-STAT and p38/MAPK signaling pathways. Activation of NF-κB and STAT3 pathway results in increase secretion of immunomodulatory molecules, for instance, IL-18 and IP-10, which recruit and suppress CXCR3^+^ T-cells and macrophages. LMP1 and IFN-γ upregulate immune checkpoint PD-1/PD-L1 synergistically, leading to suppression of anti-tumor activities. Furthermore, LMP1 induce the Warburg effect in NPC through upregulation of hypoxia-inducible factor (HIF)-1α and hexokinase 2, leading to enhance malignant transformation, tumor progression and resistance to radiotherapy. In addition, LMP1 facilitates metastasis by inducing EMT, through upregulation of Twist, Snail and SatB1 pathways, and remodeling of ECM, by induction of MMP9. LMP1 also promote angiogenesis, cell growth and cell survival through induction of pro-angiogenic factors, growth factors and anti-apoptotic proteins (85, 129). LMP-1 activated NF-κB induces cellular proliferation, EMT and metastasis by inhibition of tumor suppressing miR-203 (130).

EBNA1 promotes immunosuppression by converting naïve T-cells into Treg cells and promotes chemotactic migration of Treg cells by upregulating TGF-β1. Upregulation of the TGF-β1-SMAD3-PI3K-AKT-c-JUN axis induces the production of CXCL12 which recruit Treg cells by binding to CXCR4. On the other hand, TGF-β1 downregulates miR-200a, a negative regulator of CXCL12 by enhancing the SMAD3/c-Jun complex. CXCL12 also exerts an inhibitory effect on miR-200a *via* the c-Jun-miR-200a-CXCL12-c-JUN feedback loop (36). Enhanced CCL20 production in EBNA1-expressed tumor cells increased Tregs migration. Polarized-M2 macrophages by EBNA1 expression cells converted naïve T-cells into Tregs (86).

EBV non-coding RNAs (ncRNAs) include EBERs and miRNAs. EBERs mediate inflammatory response through interferon regulatory factor 3 (IRF3) and NF-κB signaling pathways by targeting RIG-I, leading to tumor progression. EBERs trigger MCP-1 and M-CSF which recruit macrophages into the TME and promote their differentiation into pro-tumorigenic TAMs (131, 132). EBER-1 expression was upregulated in NPC tissues, a high level of EBER-1 correlated with better prognosis in NPC patients (133). The EBV miRNA precursors are clustered in two main regions, which are Bam HI fragment H rightward open reading frame 1 (BHRF1) and Bam HI-A region rightward transcript (BART). EBV-miRNAs assist NPC progression by promotion of immune evasion, cell proliferation, inhibition of apoptosis and promotion of invasion and metastasis (134, 135).

## Targeting Tumor Microenvironment in Nasopharyngeal Carcinoma: Current and Future Perspectives

### Immunotherapy: Boosting T-Cell Immunity

NPC is a promising candidate for immunotherapy owing to its immunosuppressive property and expression of immunogenic EBV antigen. Several approaches which include immune checkpoint inhibitors and cellular-based immunotherapy have been employed to reinvigorate the exhausted immune cells in the NPC microenvironment (83) (**Table 2**). PD-1/PD-L1 axis has been exploited as a potential therapeutic target for reversing T-cells exhaustion and restoration of their anti-tumor function (136, 137). PD-1 is an immune checkpoint encoded by the *PDCD1* gene and expressed on the T-cell surface, whereas PD-L1 is expressed on tumor cells and immune cells. Studies reported expression of PD-L1 in approximately 70 - 90% of NPC tissues and its prognostic significance. The difference in interpretational methods leads to inconsistency of results, suggesting the need for further large-scale study (138–141).

PD-1/PD-L1 blockade therapies with anti-PD-1 antibodies, such as nivolumab, pembrolizumab, cemiplimab and anti-PD-L1 antibodies, including atezolizumab, avelumab and duravulumab have been implicated in immunotherapy against various neoplasms (136, 137). Anti-PD-1 and anti-PD-L1 therapies are generally safer than chemotherapy, with a lower incidence of treatment-related adverse events (142, 143). Single-agent studies involving PD-L1 antibodies showed lower overall response rate (ORR), OS and progression-free survival (PFS) when compared with studies with PD-1 antibodies, likely due to alternative binding of PD-1 to PD-L2 following blockade of PD-L1. Combination therapy of chemoradiation and PD-1/PD-L1 promote a synergistic anti-tumor immunity by enhancing host recognition, elimination of tumor cells and preventing T-cell apoptosis. For instance, a phase I trial investigating anti-PD1 antibody camrelizumab achieved an ORR of 34%, whereas a combination trial of camrelizumab with gemcitabine and cisplatin achieved an ORR of 91%. In spite of that, combination therapy exhibited a higher incidence of treatment-related adverse events when compared with anti-PD-1 monotherapy, suggesting synergistic toxicity (142, 143). There is also limited anti-tumor response rate towards PD-1/PD-L1 blockade therapy. Resistance to PD-1/PD-L1 therapy involves multiple mechanisms, including low tumor immunogenicity, T-cell dysfunction by other immune checkpoint receptors and modulation by noncoding RNAs.

Strategies to overcome the challenge include the incorporation of PD-L1/PD-1 inhibitors with other anti-tumor therapy. Ongoing clinical trials propose the combination of PD-1/PD-L1 blockade with other immune checkpoint inhibitors such as anti-CTLA-4 or anti-angiogenic agents such as anti-VEGFR inhibitors as potential therapeutic strategies for NPC (144, 145). Besides that, the identification of predictive biomarkers of PD-1 inhibitors will allow the selection of appropriate patients for PD-1/PD-L1 targeted treatment (136). Other immune regulatory checkpoints, for instance, the Gal-9/TIM-3 (HAVCR2) interaction represents a promising alternative as they are prominently expressed in recurrent NPC (20, 146).

Two approaches of cellular-based immunotherapy include adoptive immunotherapy, which is the infusion of *ex vivo* generated activated effector cells, such as EBV-specific CTLs, chimeric antigen receptor (CAR) engineered T-cells and cytokine-induced killer cells (CIKs) (147–150). An increase in the frequency of MDSCs, and immunosuppressive cytokines, such as CCL2 and CXCL10, underlie the development of resistance towards adoptive immunotherapy. Chemotherapeutic agents, such as gemcitabine and carboplatin may alleviate the resistance by limiting the expansion of MDSCs, secretion of pro-inflammatory cytokines and depletion of immune checkpoint molecules (151). A phase II study combining chemotherapy with adoptive immunotherapy using engineered EBV-specific CTLs as first-line treatment for metastatic or recurrent NPC patients yield a satisfactory result, with a 2-year OS of 62.9% (118). On the other hand, active immunotherapy or tumor vaccines aim to enhance recognition by the immune system by the delivery of tumor-specific antigens through antigen-presenting cells or viral vectors. These include LMP2 expressing DCs and recombinant modified vaccinia Ankara vaccine (MVA-EL), which has been proved to be safe and well-tolerated (152, 153). Besides targeting EBV-encoded oncoproteins, a peptide vaccine targeting non-EBV associated tumor specific antigen, four-jointed box 1 (FJX1), was also designed in a preclinical setting (154).

Further research should consider [1] large-scale studies for the evaluation of clinical efficacy and the optimization of dosage for the above therapy; [2] combination of therapies to augment the clinical responses, for example, combining immune checkpoint blockade with adoptive T-cell therapy (155–157); [3] development of personalized immunotherapy targeting neoantigens (158); and [4] localization of NPC-specific CTLs at the malignant site, for example, *via* chemokine-receptor interactions (159). Other alternatives, for example, CAR-NK, an emerging cellular immunotherapy strategy for cancer, should be evaluated in NPC, given its promising clinical efficacy in other settings (160).

### Tumor Infiltrating Immune Cells as Prognostic Markers

Immune score, established based upon the density of lymphocyte populations, is predictive of disease progression and distant metastases. High immune scores predict better OS, DFS and distant-metastasis free survival (DMFS) of NPC patients (37). Among TILs, CD3^+^, CD4^+^, and CD8^+^ T-cells as well as CD56^+^ NK cells are good prognostic factors for NPC patients (161–163). Despite its immunosuppressive role, high Foxp3^+^ T-cells to CD8^+^ T-cells ratio was associated with favorable PFS in early-stage NPC patients (164). Similarly, Ooft et al. reported Foxp3 as an independent predictor for better OS in NPC patients (165). On the other hand, CD68+ TAM density predicted favorable DFS, whereas stromal CD163^+^ M2-like TAMs correlated with poor OS and PFS in NPC patients (20, 166, 167).

Nonetheless, there are discrepancies among studies investigating the prognostic significance for different TIICs, due to different methodologies used. Thus, further studies should consider the development of a standardized method for the evaluation of TIICs as an indicator or predictor for the progression and therapeutic response of NPC patients.

### Anti-angiogenic Therapy

Angiogenesis is important to meet the huge demand for oxygen and nutrients by tumor cells, thus, anti-angiogenic therapy using angiogenic inhibitors is a promising anti-cancer therapeutic strategy. Conventional chemotherapeutic agents, for instance, epirubicin, elicits inhibitory activity on angiogenesis by inducing non-specific vascular toxicity (168). On the other hand, hypericin-mediated photodynamic therapy exerts its antitumor activity by targeting the tumor vasculature (169). Several small-molecule inhibitors of VEGFR such as apatinib are employed in angiogenesis-targeting therapy for NPC patients (170, 171). The inhibition of angiogenesis could enhance the effectiveness of standard chemoradiotherapy against NPC. Combination of VEGF inhibitor bevacizumab with chemotherapy promoted microvasculature maturation, enhanced immune infiltration and exhibited promising tumor response (172). Combination therapy of apatinib with cisplatin showed a synergistic effect in inhibition of tumor growth, repression of VEGFR-2 expression and reduction in microvascular density in preclinical models (173). On the other hand, combining apatinib and radiation enhances the anti-angiogenic effect and increases hypoxia in tumor tissues, leading to an anti-tumor effect (174). Endostatin, an endogenous angiogenesis inhibitor is another anti-tumor agent tested in clinical trials. Endostar, a recombinant endostatin, sensitized NPC towards radiation therapy by induction of endothelial cell and tumor cell apoptosis and repression of radiation-induced pro-angiogenic factors (175). Phase II multicentre randomized controlled combining Endostar therapy with standard chemoradiation reported an improved remission rate of cervical lymph node metastasis, with a slight improvement in objective response rate (121). To sum, anti-angiogenic agents targeting VEGF and VEGFR can enhance the effectiveness of chemoradiotherapy through inhibition of blood vessels formation, normalization of blood vessels, which restore vascular function, improve tumor perfusion, and drug delivery, and activation of immune response (176).

Currently, anti-angiogenic therapy faces the challenge of the development of resistance, which leads to limited survival benefits. The resistance is likely due to hypoxic events as a consequence of vascular depletion, resulting in the promotion of cancer invasion and metastasis, activation of alternative pro-angiogenic signalling pathways, VEGF-independent vascular mimicry, and increased recruitment of pro-angiogenic cells (176, 177). Studies unveiled upregulation of several proangiogenic factors, including VEGF, TNF-α, IFN-α, and basic fibroblast growth factor (bFGF), following hypericin-mediated photodynamic therapy. This indicates the need of combination therapies such as photodynamic therapies with angiogenesis inhibitors to enhance the efficacy of treatment (169, 178). Other challenges include toxicity effects, which include haemorrhage, hypertension, and thrombosis (79). Several strategies direct targeting the tumor vessels are introduced to increase the effectiveness of the therapy and minimise the side effects. Nanoparticles might serve as vectors for the delivery of anti-angiogenic drugs to the target site (79). Given the high effectivity for DNA enzyme (DNAzyme) to cleave targeted sequence with high specificity, DNAzyme also serves as a potential therapeutic agent for anti-angiogenic therapy. DNAzyme targeting VEGFR-1 mRNA significantly downregulated VEGFR-1, inhibited angiogenesis and altered the vascular permeability. No toxicity effect was observed *in vivo*, indicating anti-VEGFR-1 DNAzyme as potential drug candidates for further clinical evaluation (179).

### Targeting Hypoxic Condition in Nasopharyngeal Carcinoma

The increased oxygen demand by the highly proliferative and metabolically active tumor cells with inadequate oxygen supply from the impaired vasculature leads to hypoxia, a condition that mediates tumor progression, metastasis, and therapeutic resistance. This tumor-specific event has been studied extensively for the development of anti-tumor therapy. Several strategies targeting hypoxia are employed, which include a cytotoxic approach with hypoxic selectivity. In the hypoxic TME, hypoxia-activated prodrugs such as tirapazamine are preferentially activated into cytotoxic drugs and eliminate hypoxic tumor cells with high selectivity. The second approach direct inhibits hypoxia-inducible proteins. HIF-1α siRNA and PX-478 directly target HIF-1α and elicit anti-tumor activity. Hypoxia-modifying therapy improves the hypoxic condition in tumors by increasing the blood oxygen level through blood transfusion, carbogen, and nicotinamide treatment (76).

The combination of hypoxia-targeting therapy and radiotherapy exhibited a synergistic effect. Topotecan is reported to enhance the radiosensitivity of tumors by inhibiting topoisomerase I and subsequently downregulating the expression of HIF-1α target genes (180, 181). Nanotechnology is also employed to enhance the therapeutic efficacy of hypoxia-targeting therapy. In a preclinical study, nanoparticle delivering siRNA targeting HIF-1α significantly suppressed tumor growth in *in vivo* animal models of NPC (182). In recent studies, hypoxia-responsive nanoparticles have been designed for the selective release of drugs in the hypoxic tumor environment, thereby prolonging the bioavailability and improving the effectiveness of the drug (183). These nanomedicines are still in the early phase, their mechanism, delivery efficacy and safety in human bodies remain to be studied.

### Targeting Chemokines and Cytokines

Chemokines and cytokines are potential targets for the development of diagnostic biomarkers or therapeutic strategies. CCL5, also known as RANTES (regulated upon activation, normal T-cell expressed and presumably secreted), is a pro-angiogenic chemokine that can be detected in NPC patients’ plasma with 90.07% sensitivity and 56.34% specificity. Increased screening efficacy was observed when combining CCL5 with EBV viral capsid antigen (VCA-IgA) or EBV DNA assay. Inhibition of the CCL5 receptor, CCR5 with its antagonist maraviroc could suppress CCL5-associated migration of NPC cells (184, 185). Leukaemia inhibitory factor (LIF), a member of the IL-6 type cytokine family, is remarkably increased in the TME and NPC patients’ serum. Secreted by tumor cells and inflammatory infiltrates, LIF mediates the mTOR pathway in NPC, which leads to tumor growth and enhanced radioresistance. Elevated serum LIF in NPC patients is predictive of local recurrence and radiosensitivity. The blockade of the LIF signalling pathway through a LIF antagonist, soluble LIF receptor, or mTOR inhibitor, rapamycin, could sensitize NPC towards irradiation (186, 187).

The development of anti-tumor therapeutics targeting chemokines or their receptors need to consider [1] the optimal dosage of drugs with concern on their safety and efficacy; [2] evaluation of synergism effect when combined with conventional therapy and immunotherapy; [3] development of drugs with high affinity and high specificity; and [4] identification of tumor-specific targets, with minimal effects on non-tumor-associated cells.

### Clinical Applications of Exosomes

Tumor-derived exosomes are involved in a wide range of biological and pathological processes as well as intercellular crosstalk and thus are promising targets for the development of biomarkers and anti-cancer therapies. Exosomes are highly stable and can be detected in patients’ body fluids, such as urine, saliva, cerebrospinal fluid, and serum, and hence can be used in liquid biopsies as non-invasive biomarkers (96). Sera exosome concentrations have clinical significance and prognostic value in NPC patients, whereby a positive correlation between tumor lymph node metastasis and shorter disease-free survival was observed in NPC patients.

Nasopharyngeal brush or swab is a less invasive alternative for the collection of tumor tissue samples when compared to the conventional biopsy method. Elevation of EBV miRNAs, including mir-BART1-5p, mir-BART5, mir-BART6-5p, mir-BART17-5p, were reported in nasopharyngeal brush samples from NPC patients. miR-BART1-5p shows potential as a diagnostic indicator of NPC, with 93.5% sensitivity and 100% specificity, its expression level positively correlated with tumor progression (188).

In addition, miR-BART7 and miR-BART13 are significantly elevated in plasma specimens from NPC patients, the combination of both markers offers a 90% prediction of NPC. Furthermore, the level of miR-BART7 is associated with metastatic progression. Diminished levels of miR-BART7 and miR-BART13 were reported after radiotherapy, suggesting their potential as biomarkers for the monitoring of therapeutic efficacy (189). Post-treatment detection of circulating miR-BART17-5p is a potential biomarker of a poor prognosis (190). Plasma EBV-miR-BART7-3p showed 96.1% sensitivity and 96.7% specificity, whereas miR-BART13-3p has a sensitivity of 97.9% and specificity of 96.7% for the detection of NPC. miR-BART7-3p is a potential prognostic biomarker, whereby prominent levels of pre-treatment miR-BART7-3p in plasma indicated a higher risk of distant metastasis whereas post-treatment miR-BART7-3p is associated with short DMFS and OS (191, 192). Significant upregulation of miR-1301-3p was detected in the exosomes from early-stage NPC patients’ plasma, indicating their potential application as diagnostic biomarkers in early-stage NPC (193).

Upregulation of circulating exosomal circRNA circMYC is correlated with increase cell proliferation and resistance to radiotherapy in NPC patients. Circulating circMYC is a potential biomarker for the differentiation of radiosensitive and radioresistant patients with 90.24% sensitivity and 94.51% specificity (103). The detection of exosomal cyclophilin A (CYPA) combined with EBV VCA-IgA could increase the accuracy of diagnosis, indicating the utility of exosomal CYPA as a potential non-invasive biomarker for the diagnosis of NPC (194).

Besides serving as biomarkers, exosomes are potential vehicles for the delivery of cancer drugs to the target molecules. iRGD-tagged exosomes targeting αvβ3 integrin-positive NPC cells containing antagomir-BART10-5p and antagomir-18a showed remarkable anti-angiogenic efficacy in *in vitro* and *in vivo* NPC models (110). Exosomes are also employed as vehicles for tumor antigen in cancer vaccines, however, to our knowledge, exosome vaccines have not been tested specifically in NPC yet (195).

While exploiting exosomes as therapeutic and diagnostic tools, there are some challenges that need to be addressed. These include [1] time and cost consumption; [2] unsatisfactory yield, purity, and quality, which could affect [3] the sensitivity and specificity of assays, [4] absence of endogenous control for miRNA quantification, and [5] limited sample size in current studies, which affect the reliability of these studies (196, 197). On the other hand, several problems need to be considered when using exosomes as therapeutic vehicles or targets: [1] enhancing the specificity of therapy to prevent undesirable adverse events; [2] prolonging the half-life of exosomes after administration into the body; [3] effective and safe therapeutic dose of exosomes; [4] effective drug loading into the exosomes; [5] cost and time effectiveness in the production of bioengineered exosomes (93, 196).

Overall, exosomes play a pivotal role in the interplay communication between EBV-infected NPC cells and stromal cells, which contribute to the construction of the TME that favours tumor progression, development, invasion, and metastasis. Further investigations should consider [1] understanding the mechanisms underlying cellular crosstalk *via* exosomes, including the biogenesis and internalization of exosomes; [2] development of standard methods for scalable isolation and purification of exosomes; [3] large-scale clinical studies for the evaluation of exosome as reliable cancer biomarkers.

### Cancer-Associated Fibroblasts

The vast influence of CAFs on tumor development and progression made them promising therapeutic targets. Several strategies for CAF-directed anti-cancer therapies have been tested in preclinical settings, but only a few studies are focusing on CAFs in NPC (198). Disulfiram/copper exhibited anti-tumor activity by targeting both cancer cells and CAFs. It could induce apoptosis and inactivate CAFs by inhibiting the expression of α-SMA (199). Treatment with CAF inhibitor, for instance, Tranilast, inhibits CAFs from activating the NF-κB pathway, thus sensitizing NPC cells to irradiation (67). The targeting of CAFs is challenging as they demonstrate complexity in intercellular signaling, phenotype and source and lack of specific surface marker (198). Thus, further understanding of CAFs, with regards to their different phenotype and respective protumorigenic or tumor suppressive properties in TME is required for CAF-targeting therapies.

### NF-κB Signaling Pathway

The NF-κB signaling pathway is constitutively activated in 90% of EBV-associated NPC by EBV oncoproteins and genetic mutations. This pathway plays a pivotal role in the intercellular communication and regulation of immune cells in the TME, which renders it a promising target for anti-cancer therapy against NPC (200–202). Strategies targeting the NF-κB signaling pathway include anti-inflammatory compounds such as aspirin, inhibition of IκB kinase (IKK) and proteasome inhibition (130, 203, 204). Acting as an NF-κB inhibitor, aspirin reverses LMP1-induced EMT by suppressing NF-κB-exosomal secretion of LMP1 and promoting miR-203 expression (130). Inhibition of lung metastasis by aspirin is also observed in mouse models. PS1145, an inhibitor of IKK, could abrogate the NF-κB signaling pathway and subsequently inhibit the production of pro-inflammatory cytokine and cell proliferation. PS1145 significantly inhibits the tumor growth of NPC in *in vitro* cell lines and *in vivo* xenograft models (203). Bortezomib, a proteasome inhibitor, targeting STAT1 and NF-κB, could relieve the immune tolerance in NPC (204). Vitexin, a natural flavonoid glycoside targeting NF-κB, displayed promising anti-tumor activity against NPC in preclinical studies (205). Restoration of Ras-like estrogen-regulated growth inhibitor (RERG), an NF-κB inhibitor, by 5-Aza-2’-deoxycytidine and trichostatin A attenuated ERK/NF-κB signaling pathway, resulting in the inhibition of tumor growth and angiogenesis *in vivo*. Therefore, RERG might be employed as a target molecule in cancer therapy (206).

Whilst translating these bench findings into bedside application, several issues need to be addressed. As the NF-κB signaling pathway regulates multiple physiological processes, the development of therapeutic tools should consider developing drug delivery strategies with high specificity to prevent undesirable adverse events. It is reported that the NF-κB complex p50/p50/Bcl3 is prevalent in NPC but seldom found in a normal cell. Thus, Bcl3 inhibitors may represent promising therapeutic agents against NPC (202, 207). Other than that, the route of administration and dosage of NF-κB inhibitors should consider their bioavailability and safety.

## Concluding Thoughts

The advances in multi-omics technology allow researchers to unravel the complex intercellular communication in the NPC microenvironment, which contributes to the growth, development, progression, and metastasis of this malignancy. Nevertheless, there is a lack of thorough studies on the players in the NPC microenvironment, particularly, B-cells, NK cells, cancer stem cells and the ECM. Of note, spindle-shaped NPC cells predominantly located at the invasive margin of the tumor site display stem cell-like properties and are significantly associated with EMT. Further studies targeting these neoplastic spindle cells might shed light on the understanding of the mechanism underlying tumor cell dissemination, and thus facilitating future development of predictive biomarker and preventive medicine for NPC metastasis (72, 208, 209). Other than that, it is suggested that future studies look into the spatial heterogeneity of the NPC microenvironment to gain further insights into tumor heterogeneity and discover new opportunities for the development of theragnostic tools (210).

Several anti-cancer drugs targeting TME have been tested in clinical trials, however, several pre-clinical and clinical barriers remain to be overcome. Preclinically, models for cancer drugs are inadequate to visualize the complexity of TME. Cell and tissue-engineered models with 3-dimensional co-culture systems could be utilized to recapitulate the cellular organization, growth kinetics, cellular heterogeneity, intra- and intercellular interactions *in vitro* to improve translation and reduce animal testing (211). Clinically, given the inter-patient heterogeneity in genetic and epigenetic factors, responses towards drugs are highly variable among individuals. Thus, an optimal combination of therapeutic strategies should be designed based on the integration of individual information on TME landscape, genomic and molecular profile to ensure precision, safety, and effectiveness of cancer therapies, which ensure better health outcomes of NPC patients. Moreover, to reduce the adverse events of therapeutic and enhance therapeutic efficacy, strategies to improve specificity in targeting, delivery, and release of drugs against TME should be considered. These include the engineering of nanoparticles or exosomes with specific ligands as vehicles for the delivery of drugs into the target cells. Several delivery systems which could enhance the effectiveness of delivery have been developed, which include arginine-modified hydroxyapatite nanoparticles and fusion-based NPC-specific lipid nanoparticles (212, 213).

On the other hand, there is a need for the discovery of tumor-specific molecules as targets for the specific delivery of therapeutic agents or disease diagnosis *via* liquid biopsy. The biomarker should have high specificity, sensitivity, accuracy, precision, and reliability, inexpensive and timely. While diagnostic biomarkers allow the early detection of NPC, prognostic biomarkers will predict the disease progression of patients and facilitate the development of prophylactic drugs. Advancement in molecular technologies, together with enormous databases integrating molecular and clinical data sets, will accelerate the research findings and their translation into clinical use.

In conclusion, the NPC microenvironment consists of cellular and acellular players which serve as targets for the development of therapeutic, diagnostic, and prognostic tools. Despite that, most of them are only tested in preclinical or early phases of clinical studies. Hence, large-scale clinical studies should be considered to evaluate the reliability of TME components as diagnostic tools, and guidelines or standards should be developed to ensure safe clinical use of therapeutic tools.

## Author Contributions

ZS, PS, and S-CC were involved in the conceptualization and design of the content. ZS wrote and revised the manuscript. PS, C-OL, and S-CC reviewed and revised the manuscript. All authors have read and agreed to the published version of the manuscript.

## Funding

This study was supported by grant from the Ministry of Higher Education (MOHE) through Fundamental Research Grant Scheme (FGRS/1/2020/SKK0/UCSI/02/2) and UCSI University Research Excellence and Innovation (REIG-FMS-2020/009).

## Conflict of Interest

The authors declare that the research was conducted in the absence of any commercial or financial relationships that could be construed as a potential conflict of interest.

## Publisher’s Note

All claims expressed in this article are solely those of the authors and do not necessarily represent those of their affiliated organizations, or those of the publisher, the editors and the reviewers. Any product that may be evaluated in this article, or claim that may be made by its manufacturer, is not guaranteed or endorsed by the publisher.
